# Drivers of Antibiotic Use in Poultry Production in Bangladesh: Dependencies and Dynamics of a Patron-Client Relationship

**DOI:** 10.3389/fvets.2020.00078

**Published:** 2020-02-28

**Authors:** Abdullah Al Masud, Emily Kate Rousham, Mohammad Aminul Islam, Mahbub-Ul Alam, Mahbubur Rahman, Abdullah Al Mamun, Supta Sarker, Muhammad Asaduzzaman, Leanne Unicomb

**Affiliations:** ^1^International Centre for Diarrhoeal Disease Research, Bangladesh, Dhaka, Bangladesh; ^2^Centre for Global Health and Human Development, School of Sport, Exercise and Health Sciences, Loughborough University, Leicestershire, United Kingdom; ^3^Paul G. Allen School for Global Animal Health, Washington State University, Pullman, WA, United States; ^4^Centre for Global Health, Institute of Health and Society, University of Oslo, Oslo, Norway

**Keywords:** small-scale commercial poultry farms, antibiotics, credit, dependencies, patron-client relationship, antimicrobial resistance

## Abstract

**Background:** There is increasing concern around the use of antibiotics in animal food production and the risk of transmission of antimicrobial resistance within the food chain. In many low and middle-income countries, including Bangladesh, the commercial poultry sector comprises small-scale producers who are dependent on credit from poultry dealers to buy day-old chicks and poultry feed. The same dealers also supply and promote antibiotics. The credit system is reliant upon informal relationships among multiple actors as part of social capital. This paper aims to describe dependencies and relationships between different actors within unregulated broiler poultry production systems to understand the social and contextual determinants of antibiotic use in low-resource settings.

**Methods:** We used a cross-sectional qualitative design including in-depth interviews among purposefully selected commercial poultry farmers (*n* = 10), poultry dealers (*n* = 5), sales representatives of livestock pharmaceutical companies (*n* = 3) and the local government livestock officer as a key-informant (*n* = 1). We describe the food production cycle and practices relating to credit purchases and sales using social capital theory.

**Findings:** Poultry dealers provide credit and information for small-scale poultry farmers to initiate and operate their business. In return for credit, farmers are obliged to buy poultry feed and medicine from their dealer and sell their market-ready poultry to that same dealer. All farms applied multiple antibiotics to poultry throughout the production cycle, including banned antibiotics such as colistin sulfate. The relationship between dealers and poultry farmers is reciprocal but mostly regulated by the dealers. Dealers were the main influencers of decision-making by farmers, particularly around antibiotic use as an integral part of the production cycle risk management. Our findings suggest that strategies to improve antibiotic stewardship and responsible use should exploit the patron-client relationship which provides the social and information network for small-scale farmers.

**Conclusion:** Social capital theory can be applied to the patron-client relationship observed among poultry farmers and dealers in Bangladesh to identify influences on decision making and antibiotic use. Within unregulated food production systems, strategies to promote the prudent use of antibiotics should target commercial feed producers and livestock pharmaceutical manufacturers as a first step in developing a sustainable poultry value chain.

## Introduction

Demand for animal sourced protein for human consumption has increased from 7 to 25 g per capita per day between 1960 and 2013 (1) due to increased access to information on meeting nutritional requirements and higher purchasing ability in low and middle-income countries (LMICs) (2).

In Bangladesh, ~37% of all animal protein meat consumption originates from poultry (3). Small-scale commercial broiler poultry farms, defined as those having <5,000 birds in each batch (4), comprise 81% of the commercial poultry sector (3) providing about 78% of the total poultry meat supply in Bangladesh (5). Small-scale broiler farms are typically traditional open system broiler houses with natural ventilation, manual feeding and open-sided walls. They are usually built on the land surrounding the homestead with locally available low-cost materials and often rely on family labor (6).

These poultry farmers have limited working capital, poor access to formal credit and also have limited information on market opportunities or new technologies (7). For example, small-scale commercial poultry farmers cannot afford to buy day-old chicks and poultry feed directly from the hatcheries or feed manufacturing companies and thus depend on dealers or agents who provide a credit system (8). This credit system in turn provides social networks and relationships, described in social capital theory, through which information can flow. For example these networks can convey useful information about opportunities and choices, as well as information about market needs (9, 10).

Social networks can also affect decision making, influenced through the credit system (9). Within the food value chain, there are multilevel relationships among different actors which can be defined as social capital captured in the form of social relationships with expected returns, either formal or informal, generated by individuals in their interaction with other individuals (11). Social capital can also facilitate access to information, allowing the coordination of activities, and ultimately influencing decision-making (12).

There is increasing concern around the use of antibiotics in animal food production and the risk that poses antimicrobial resistance transmission in the food chain (13). In small-scale commercial broiler farms in Bangladesh, antibiotics are more likely to be used without veterinary supervision for therapeutic purposes. Antibiotics are also used in sub-therapeutic doses by adding them to feed and water for prophylaxis, growth promotion and as a risk-management strategy (4). Sub-optimal antibiotic prescribing and use is prevalent in both human medicine and livestock production as a means of managing risk (14). In Bangladesh, the Animal Feed Act prohibits the use of antibiotics in feed (15). However, anecdotally poultry farmers circumvent the law by including antibiotics in drinking water provided to broilers. Moreover, governance and monitoring of small-scale producers is weak.

The scientific community assigns both social context and entrepreneurs' behavior a central role in the growth and development of the global economy. However, the relationships between these two factors have not been sufficiently studied in business and economics (9). Few studies have examined the social and economic drivers of antibiotic use in unregulated food production systems. As part of a study to determine drivers of antibiotic use in poultry production, this paper aims to describe the nature of dependencies and reciprocal relationships between different actors in small-scale broiler poultry production (social networks) and how the dependent relationships influence farmers' decisions to use antibiotics to raise broilers. We aimed to examine the social and contextual determinants of antibiotic use in low resource settings where antimicrobial resistance poses increasing health risks to socially and economically disadvantaged populations.

## Materials and Methods

### Study Area, Study Participants, and Sample Size

Small-scale non-intensive poultry farming is predominantly located in rural and peri-urban areas of Bangladesh. Our study was conducted in a rural area of *Mirzapur Upazila* (sub-district) under *Tangail* district, ~70 km from the capital city, Dhaka, where the poultry industry constitutes a significant part of the local economy. *Mirzapur* has a total area of 373.88 km^2^ and has a population of 423,708 (16). Small-scale poultry farms in this area were made of rudimentary housing structures with bamboo or concrete or mud floors which were covered with rice bran as bedding. Walls were typically built with tin and chicken wire for ventilation. A plastic cover on top of the chicken wire, could be folded up or down for protection (17).

We used cross-sectional qualitative methods. We used purposive sampling to enroll participants for face-to-face in-depth interviews. We collected a list of broiler poultry farms and identified 10 small-scale commercial poultry farms (<5,000 birds) from different unions within *Mirzapur Upazila* and interviewed farm workers (*n* = 10) in their poultry premises. We asked poultry farmers to name some leading livestock pharmaceutical companies who had been marketing their products in the local market. We also collected mobile phone numbers of the sales representatives of the respective companies from the farmers. We ranked top three pharmaceuticals companies from the list and communicated them by phone. We interviewed sales representatives (*n* = 3) in their convenient time and locations within their working areas. We conducted in-depth interviews with poultry dealers (*n* = 5) representing each of the top five poultry feed brands cited by farmers. Poultry dealers supply day-old chicks, poultry feed, and medicines. They also purchase mature market-ready birds from the poultry farmers.

Additionally, the local sub-district *(Upazila)* government livestock officer was interviewed as a key-informant (*n* = 1) in the local government livestock office. The livestock officer was a government recruited qualified veterinarian with responsibility to ensure the deployment of government poultry development policies such as; formation of poultry smallholder groups, community based organizations and producer associations, quality control of poultry feeds, and feed ingredients, supervision of the veterinarian prescribing antibiotics and other medications, registration of all commercial poultry farms with the department of livestock services, support in planning and implementation of all livestock related extension activities at the grass-root level and ensuring biosafety and biosecurity on farms.

### Data Collection

Data collection was performed during July–September, 2017 by a trained single researcher who had a social science background. In-depth and key-informant interview guidelines were developed based on the research objectives and relevant literature. In addition, a checklist was developed for use for spot observations to record medicines, supplements, additives used together with poultry feed products on the farms. Both interview guidelines and checklist for spot observation were field-tested before implementing the study. Interviews were conducted after obtaining informed consent from the study participants. Data collection was carried out in Bengali language. Interviews with participants were audio-recorded and field notes used for additional information. Medicines used by farmers were photographed to help complete the checklist. During analysis, a veterinary physician categorized all the items into antibiotic and non-antibiotic medicines.

### Data Analysis

Anonymized transcription of audio-recordings was carried out in Bengali language. Based on our literature review and research questions, we used a thematic data analysis, which is a systematic approach to data analysis used to analyze classifications. This provides the opportunity to code and categorize data into themes; present themes (patterns) that relate to the data; illustrate the data in great detail, and deals with diverse subjects via interpretations. In addition, the thematic analysis allows the researcher to determine precisely the relationships and coherence between concepts (18). In thematic analysis, we used both inductive and deductive approaches (19). We prepared deductive codes based on research questions and existing literature and inductive codes identified from the interview transcripts. Transcripts were coded using both deductive and inductive approaches and compiled in MS Excel. We followed the recommended steps of thematic approach namely: data familiarization; generating initial codes; searching for themes; reviewing potential themes; defining and naming themes and producing the report (19). To check the coding reliability, two individual researchers independently coded the same data and reviewed the codes applied.

We described findings under key components of social capital theory; information flow and decision making. Additionally, a conceptual framework for the patron-client relationship between farmers and poultry dealers was developed. We reached data saturation within the sample size.

## Results

### Characteristics of Study Participants

The poultry farmers were male, aged between 20 and 47 years and had worked in poultry farming for between 1 and 24 years. Among the 10 farmers, 8 had high school education. For all participants, poultry farming was the main income source with some supplementary income from seasonal crops. The majority reported a monthly income between 10,001 and 20,000 Taka (US$ 121–241) from their poultry business. The flock size of the farms ranged from 500 to 1,300 birds on the day of interview. Interviewed poultry dealers had been in the trade from 7 to 17 years and their age was between 39 and 47 years all being male. Three out of 5 dealers had completed secondary education. The pharmaceutical sales representatives were male, completed graduation from university and had been in the field of livestock medicine marketing from 2 to 5 years. The sub-district *(Upazila)* government livestock officer who interviewed as a key-informant was male and had been working in the *Mirzapur* sub-district for 2 years.

### Role of Farmers and Poultry Dealers/Credit Based Marketing

Poultry farmers reported that while starting up their farm, they usually consulted with other farmers and poultry dealers to determine the estimated cost required for installation of poultry sheds and equipment. Most interviewed farmers started poultry production with only a poultry shed/pen and some basic equipment such as; water feeders, feeding tray and brooders (heating equipment). They usually bought day-old chicks, poultry feed, medicines and additives from a poultry dealer based on their personal relationship and a credit facility. Poultry dealers reported that they also informed new farmers about the support that they can provide (e.g., credit purchase, selling poultry). “*Few years back, I have shown my interest to start farming and consulted with a dealer. He said, if I feel encouraged enough, then he will give full support to start a poultry farm. Then I started my farm and purchased everything on credit from dealer.”* [Commercial poultry farmer, age-35].

The key-informant reported that dealers often impose some preconditions to the credit receivers such that the farmers are bound to buy all feeds and medicines from that respective dealer for the entire period of poultry nurturing and that the farmers sell all mature, market-ready poultry to that respective dealer. “*If you receive any favor from any person, you will naturally have a good impression about that person and take his advice.”* [Livestock officer of sub-district].

Farmers reported that they did not have many options when choosing poultry feed, because it was determined by what was offered by their respective poultry dealer. Poultry feed was also obtained on credit. “*I started my business with a small capital and purchased day-old chicks and feed from a dealer on credit…Therefore, we have no opinion or option to choose… We purchase whatever they have and whatever they suggest. We repay our credit by selling all ready-for-sale poultry to them.”* [Commercial poultry farmer, age-28].

Poultry dealers reported that when they started their business, they sold a range of poultry feed brands. Based on the comparisons among different brands and feedback from poultry farmers, dealers decided on the most popular and profitable brands to market; feed companies also provided dealers with reasonable credit facilities for their business. “*First, I started my business with some different brands. Finally, I tied up with a particular brand, when they assured me that they will provide products with highest credit facilities, because we sell most of the products on credit.”* [Poultry dealer, age-40]. Poultry dealers reported that they have direct interactions with poultry hatcheries, poultry feed and pharmaceutical manufacturing companies. Feed companies give credit purchase facilities by fixing a sales target for one poultry dealer, who acts as the sole distributor of that feed brand in one *upazila*. For example, one dealer reported that 50 tons (1,000 bags) of poultry feed was the sales target for 1 month. If a dealer achieves the sales target, they receive Taka 20–25 (US$0.23–0.29) per bag of feed sold. The amount of commission is around US$250 to 300 per month for 50 tons of feed. Poultry dealers also reported that for renowned and popular medicine brands, they usually pay cash, but a credit facility is also available for up to 30 days. If dealers pay cash for medicines instead of credit, they get different percentages of commission for different products. “*As we sell everything on credit to the commercial poultry farmers, we therefore prefer credit purchase from the medicine company.”* [Poultry dealer, age-40].

Both dealers and farmers reported that credit facilities, both for sales and purchase, depend on “a good relationship” which they consider a bilateral requirement that stands upon “trustworthiness” and previous “healthy” financial transactions. The key-informant reported that over the last 10 years there has been an increasing trend of credit-based business established through the entire supply chain to sustain a competitive market. Feed and pharmaceutical manufacturers often provide credit facilities to the dealers so that they can expand their business.

### Role of Pharmaceutical Company Sales Representatives

Sales representatives are another group participating in the broiler production social network. They reported that the pharmaceutical company set sales targets. The target varied by season, but the average sales target was Taka 600,000−800,000 (~US$ 7,000–10,000) per month. There were peaks in demand for antibiotics by season for poultry and other farm animals. “*We are often given some targets. In the case of poultry, the target is higher during winter when disease occurrence is higher than other months. We are bound to achieve the target”* [Pharmaceutical sales representative, age-31].

Sales representatives reported that they perform a range of activities to achieve their sales targets. They arrange workshops for the farmers and dealers to provide basic information about the application of different medicines including antibiotics. Sales representatives visit government staff including *Upazila* livestock officers, *Upazila* veterinary surgeons, veterinary field assistants, and poultry dealers on a regular basis and brief them on the latest products and provide promotional offers or incentives. “*The company will never give you any target that is easily achievable. Meeting targets mostly depends on how we can motivate veterinarians to prescribe our medicine.”* [Pharmaceuticals sales representative, age-30].

Sales representatives reported that feed companies, pharmaceutical companies and hatcheries provide farmers with veterinary treatment service for their broilers free of cost. Sales representatives facilitate these services to the farmers through the poultry dealers. Sales representatives reported that “a good relationship” with dealers and veterinarians often helps them achieve sales targets.

### Information Flow- Farmers' Knowledge on Antibiotic Application for Poultry Raising

Some poultry dealers reported that they learn how to treat common diseases by following veterinarians' prescriptions. Others learned through feedback from other farmers after applying antibiotics for a specific disease. Dealers apply this combined knowledge whenever any farmer reports concern over poultry disease to them. If the antibiotic does not work or the disease spreads rapidly, then they ask veterinarians from the feed or pharmaceutical company for possible solutions. “*About 80% of poultry farmers maintain their poultry by gathering experience from dealers and sales representatives. Only 20% of farmers consult with the veterinarians when they are unable to control disease and death of poultry.”* [Pharmaceuticals sales representative, age-30].

The key-informant reported that in most instances, sales representatives also provide treatment advice directly to the farmers. Sales representatives reported that pharmaceutical companies provide product manuals for distribution among poultry dealers, which list symptoms of the disease and the appropriate treatment. Small-scale commercial poultry farmers were able to identify very few medicines as antibiotics among all medicines that they apply everyday to poultry; instead they explained the purpose of using medicines. “*We describe the symptoms of the disease to the dealers or sales representative and they suggest medicine accordingly.”* [Commercial poultry farmer, age-28].

### Decision Making- Antibiotic Application for Broiler Production

**Figure 1:**
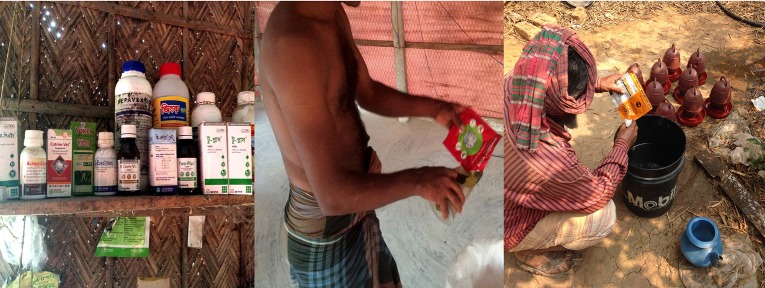
Application of antibiotics in poultry feed and drinking water.

Spot observations and interviews showed that all commercial poultry farmers used antibiotics throughout the poultry production cycle (Table 1, Figure 1). The most common antibiotics observed at the time of interview were ciprofloxacin and doxycycline (7/10 farms), followed by tylosin and doxycycline (combination), oxytetracycline, enrofloxacin, and erythromycin (6/10 farms). Colistin sulfate was used by 5 of the 10 surveyed farms. Multiple antibiotics were used on the same farm.

**Table 1:** Antibiotics used in small-scale commercial poultry farms during spot-checks.

**Generic name**	**Number of farms using antibiotics (*N* = 10)**
Ciprofloxacin	7
Doxycycline	7
Tylosin + Doxycycline	6
Oxytetracycline	6
Enrofloxacin	6
Erythromycin	6
Amoxicillin	5
Colistin sulfate	5
Levofloxacin	4
Erythromycin + Trimethoprim + Sulphadiazine	4
Gentamicin + Enrofloxacin	4
Telcomycin	4
Sulphamethoxazole + Trimethoprim	3

Administration of antibiotics started from day 1 and continued until sale of mature live birds, based on recommendation by dealers. Table 2 summarizes the antibiotics administered routinely, according to the age of poultry and the reasons given by farmers and dealers for their use. “*I use medicine routinely for the entire period from day one to the last day to prevent my poultry from disease and for better growth.”* [Commercial poultry farmer, age-45]. Use of antibiotics was therefore for prevention of diseases in some cases. Poultry dealers and farmers reported that some diseases occur seasonally; hence different antibiotics were applied in each season. For example, *Gumboro* (a highly contagious acute viral infectious disease in chickens) occurs at the end of the rainy season (August/September). Dealers reported that, during winter (December to February), farmers fear “bird” (avian) flu and therefore apply antibiotics for prevention. This was confirmed by the poultry farmers who reported that they apply antibiotics more during winter compared to other seasons, because winter is the bird flu season. Farmers reported that they fear epidemics and said once a disease breaks out; it is difficult to save the flock. If any poultry appear unwell, farmers apply antibiotics to all birds. During spot observations we found a wide spectrum of antibiotics that farmers had used for disease prevention in all farms (Table 2).

**Table 2:** Antibiotics routinely used in small scale commercial poultry farms reported during in-depth interviews.

**Time of antibiotic application**	**Generic name of the antibiotic**	**Reported reason for use**
Within first 10 days	Amoxicillin	To prevent bacterial infections
Within first 10 days	Endocyn	To prevent fungal infections
Anytime but especially in first 10 days	Oxytetracycline Hydrochloride	Growth promotion
Second 10 days	Doxycycline	To prevent respiratory disease
Day 18–20 (high use during winter)	Erythromycin Thiocyanate, Sulfadiazine Sodium, Trimethoprim composition	To prevent flu and cold
During rainy season	Ciprofloxacin	To prevent *Gumboro* (Highly contagious acute viral infectious disease in chickens)
When one or two poultry identified with symptoms	Ciprofloxacin	To prevent watery lime feces

Pharmaceutical sales representatives reported that the reliance on antibiotic use is higher among small poultry farmers who have limited capital, small investment and receive substantial credit from the dealer. “*Small and medium-scale poultry farmers use more antibiotics because they have small capital and huge credit, therefore they do not want to take any risk.”* [Pharmaceuticals sales representative, age-31].

### Patron-Client Relationship

**Figure 2:**
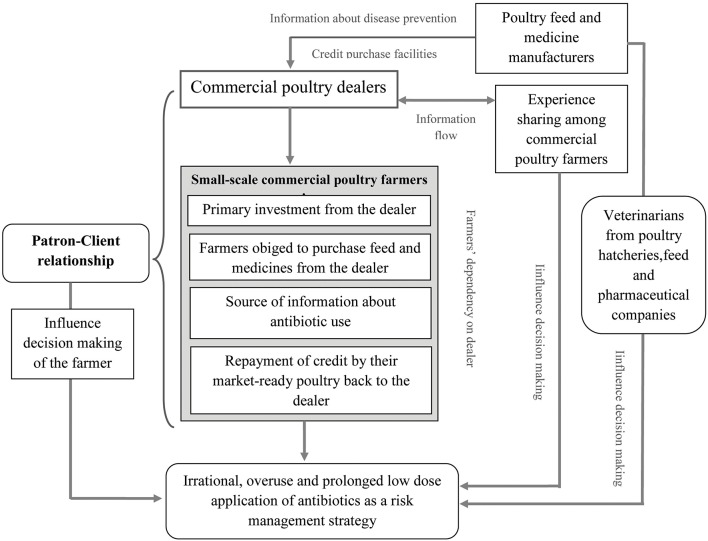
Factors that influence farmers' decisions on antibiotic use in broiler production.

The roles of farmers, dealers, and pharmaceutical company sales representatives in poultry rearing were intimately linked; these data were used to develop a conceptual framework of the patron-client relationship (Figure 2). Dealers invest in the poultry farms from start up through credit purchase with preconditions that the farmers buy all feed and medicines from them for the entire production cycle. Farmers are also obligated to sell the mature, market-ready poultry back to the same dealer. The credit arrangement therefore provides an unwritten contractual agreement between the credit receivers (poultry farmers) and a specific dealer. The dealers control information flow from pharmaceutical companies regarding disease prevention and growth promotion. In addition, they provide information of the retail market about how to maximize the farmer's return on investment. Both the information about disease prevention and market information impact farmer's decision making.

The key informant defined this relationship between dealers and poultry farmers as a “gentleman's agreement,” where both parties protect their own benefit “*The relationship among poultry dealers and poultry farmers seems very harmless. That's why I say it is a ‘gentleman's agreement', where both dealers and farmers look for their own benefits and never complain about each other. The relationship stands on a gentleman's agreement so the government and any authority can't get access to this relationship.”* [Livestock officer of sub-district].

Both the key informant and farmers reported that when there is an adverse outcome (such as: mortality, epidemic, low bird weight) the farmer bears the full cost “*We buy everything on credit from the dealer. If I face any loss of my business, I will alone be liable for that.”* [Commercial poultry farmer, age-45].

We asked poultry dealers about how they recover money from farmers if they face losses. Poultry dealers said that they often extend the credit facility to farmer so that they can purchase necessary commodities for another batch of broilers to overcome the previous loss. “*All batches are not equally profitable for a farmer. Sometimes they face loss. In that situation we sometime give another credit to overcome the losses. Because, we also depend on the farmer, if farmers live then we live.”* [Poultry dealer, age 40]. Farmers reported that when they sell mature, market-ready poultry to the dealer, the amount in credit is deducted and they receive the remaining amount.

The key informant compared this dealer-farmer relationship to a micro financing institute and credit receiver relationship, whereby dealers have a long-term business plan similar to that used by micro-financing institutes but in a different format. If a farmer gets a profit from the first broiler batch, then he will take a larger amount in credit from dealers for a second batch to expand the farm and maximize profit. On the other hand, if farmers make a loss, they make all the next purchases on credit to recover from the loss. This credit system is an integral part of the poultry food value chain, where dealers also get support from the poultry hatchery; poultry feed manufacturer and pharmaceutical companies.

## Discussion

Our study revealed a critical key relationship between poultry dealers and farmers in the provision of credit to farmers that is essential for their livelihoods and a critical driver for antibiotic use during poultry rearing. Poultry dealers act as the sole supplier of a single poultry feed brand, receive credit, and commission from poultry feed suppliers and manufacturers, and obtain antibiotics on credit from pharmaceutical company representatives. Dealers create functional connections between the input producers and the small-scale farmers by sourcing the production inputs from the large companies through their networks and self-credibility. They purchase production inputs on credit and distribute it to farms and purchase harvested products from the small farmers, thus keeping the production cycle moving (20). Pharmaceutical representatives always focus to achieve the sales target and maintain a continuous liaison with dealers. Pharmaceutical companies provide their own veterinarian's support through dealers to the small farmers. There is a substantial penetration and promotion of animal drug use by pharmaceutical companies and their agents (21). Social science research on antimicrobial resistance has revealed how antibiotic use in livestock and food production has been driven by the global pharmaceutical industry more than the practices and knowledge of end-users, such as farmers (22).

This study found that farmers routinely used antibiotics, including those banned for use in animals such as colistin. Multiple antibiotics were applied from day 1 of the production cycle until the point of sale. Farmers use antibiotics ubiquitously for growth promotion and prophylaxis as a risk-management measure (4). The supply chain of antibiotics distributes antimicrobial products to the wholesalers, retail pharmacies and feed dealers and even to commercial poultry farmers. Most commercial poultry farmers use antibiotics themselves directly from feed dealers or even directly from companies (23). Other studies show that most farmers (>60%) used antibiotics without the prescription of the veterinarians (24). Another study in Bangladesh among small-scale layer farms of *Mymensingh* district revealed that almost all (94%) farmers were using antibiotics without maintaining the minimum withdrawal period before marketing (25). Residues of ampicillin, ciprofloxacin and enrofloxacin have been detected in liver and meat of broilers (26). The presence of antibiotic residues in meat poses further risks to human health (27). Similar to our findings, antibiotics were considered an essential element for disease prevention and treatment in Cambodia, where farmers focused on the benefits to their food production system rather than concerns about the consequences of antibiotic use (28). Prophylactic use of antibiotics in commercial poultry production is also common in other countries in Asia including Cambodia, Indonesia, Vietnam, and Thailand (29). Poultry farmers using antibiotics as a growth-promoters perceive the costs of antibiotics to be paid off by rapid growth rates (30). Using more, or different types of antibiotics is also perceived by farmers as keeping their farm more secure and productive (24).

Our study found that the major poultry production inputs are purchased from dealers on credit. Dealers control the information flow to farmers and, in the absence of any other sources of information, farmers follow these recommendations without question. In contrast, poultry farmers from central Uganda were found to make decisions about antimicrobial use themselves; either based on previous experience, with guidance from dealers or from previous veterinary advice (31). Despite concerns around antibiotic use in food production, the growing poultry sector in Bangladesh is highly valuable to the economy through the creation of direct and indirect employment opportunities, improved food security and increased access to animal-source protein (3).

As described in social capital theory, social networks include a range of relationships that can offer access to production resources that include information, credit purchase facilities, physical or human capital. This in turn affects decision-making processes and collective action through reciprocity and mutual trust (9). Important social networks have been highlighted among small broiler farmers in a high income country setting, but in this case, the social networks involve farmers' cooperatives that have ready access to qualified veterinary practitioners and technicians, who share the decision-making with farmers, and empower the farmers with greater autonomy through knowledge-sharing (32). Due to limited capital and market information channels, we found that small-scale farmers are highly dependent on dealers for the entire process of poultry production and marketing in a credit based reciprocal relationship. Dealers, who obtain income through sales commission, forcedly push so-called nutrient-ready feed and antibiotics through credit facilities and farmers are economically tied to buy chicks and feed from same dealers (8). These farmers face the challenges of limited access to institutional credit, inadequate knowledge of poultry rearing practices, loss of profit due to death or disease outbreaks among flocks. They have no access to production and marketing information or poultry marketing channels (33).

Our findings also resonate with anthropological theory such as Social Lives of Medicine which proposes medicines as important political and economic actors within society (34), In viewing antibiotics as “things” they become commodities that have their own social life and consequences, aside from their medicinal or biochemical properties (34). In broiler poultry farming they play a particularly salient role in both the transactional aspects of antibiotics, and the symbolic meaning attributed to antibiotics. Anthropological approaches also highlight that professional practices of veterinarians and animal technicians are shaped by their economic value within a given market. These professions now have to develop economic models that do not rely on drug use. Anthropological and social science approaches can play an important role in developing alternative economic models and reducing antibiotic use in livestock production (22).

In many developing countries, a large proportion of the broiler poultry farms benefited from contract farming (6) which provides day old chicks, feeds, veterinary services, and technical supports on credit and ensures purchase of the output (35). Vertically-integrated poultry contract farming was introduced in Bangladesh in 1994 by a single company as an experimental extension program and later, a limited number of companies initiated contract farming (7). However, the growth of contract farming has been very slow and is still undertaken by only a small number of companies. Self-regulated small-scale broiler farming still dominates the broiler industry (6). A disorganized marketing system prevails in Bangladesh that further disadvantages small farmers. A multi-country systematic review highlights that most small-scale commercial poultry farms in LMICs rely on an informal marketing system based on verbal contracts to sale-purchase agreements. In this verbal agreement, farmers are less likely to have the choice of technology, input supplies or any service support (36). The influence of social capital on the decision making for the small and medium entrepreneur is reasonably important because for the access to production resources and market information. Therefore, the decision is mostly carried through the social networks (37, 38).

In Bangladesh, the use of antimicrobials for sub-therapeutic purpose (growth promotion) has been prohibited (15). However, research has revealed antibiotic residues are still present in poultry meat and eggs. Low veterinary regulation and low enforcement of the Act may be the root cause of this situation (23). Food safety administration and inspection does not include the monitoring the entire chain of production (39). Likewise a multi-country study suggested that poor state governance is associated with less effective controls of antibiotic use in the human and animal sectors (40). In most instances, the absence of a clear legislative framework on the use of antimicrobials in livestock production in LMICs may result in increased irrational consumption (1). Other factors that adversely impact the capacity to control antibiotic use in food animals include limited technical staff in agriculture departments to monitor farming practices as well as over-the-counter availability of antibiotics (28).

Some limitations in our study include the inclusion of broiler farms from only one region of Bangladesh, although we consider commercial poultry farming in this area to be similar to most areas of the country. We also interviewed a limited number of key informants and pharmaceutical company representatives. Further research is needed to gain qualitative insights into ways of engaging farmers, dealers and agents in antibiotic stewardship initiatives, to improve biosecurity of small-scale production systems, and reduce the financial risk to farmers of reducing their reliance on antibiotics.

In conclusion, in a setting where governance is weak, relying on law enforcement to reduce antibiotic use in livestock farming is inadequate. Investigating ways to exploit the patron-client relationship for improved antibiotic stewardship is a logical next step in developing a sustainable poultry value chain (4). Regulation, monitoring, and control programs for the prudent use of antibiotics in food-producing animals must begin with feed manufacturers and small/medium-scale poultry industries and must address or exploit the strong bonds within the patron-client relationship.

## Data Availability Statement

The datasets generated for this study are available. Original data are available at: https://doi.org/10.5285/630759ac-b0ca-4561-8eec-414b47e14829.

## Ethics Statement

The study was approved by the Institutional Review Board (IRB) of icddr,b (protocol number PR16071) and Loughborough University, UK (R17-P037). Prior to taking part, all participants received information about the study (verbally and in writing) and signed consent forms for interview and for the publication of any photographs taken.

## Author Contributions

ER, MI, and LU conceptualized the study, secured funding, conceived, and designed the original protocol. All authors were involved in designing the data collection instrument, and selection of field sites. AMas developed data collection instruments and supervised the data collection. AMas, AMam, and SS analyzed and summarized the data. AMas led the writing of the first draft of the manuscript with LU and ER. MI, M-UA, MA, and MR provided input on data interpretation and analysis. LU guided the writing as the senior author and all authors contributed to writing the first draft of manuscript. The authors are grateful to the study participants for providing their time and invaluable information.

### Conflict of Interest

The authors declare that the research was conducted in the absence of any commercial or financial relationships that could be construed as a potential conflict of interest.
